# Supplementary material for: Characterization of Potency of the P2Y13 Receptor Agonists: A Meta-Analysis
Source: Int J Mol Sci. 2021 Mar 27;22(7):3468. doi: 10.3390/ijms22073468 (PMC8036966; doi:10.3390/ijms22073468)
Supplement: Supplementary file 1 [file ijms-22-03468-s001.zip › Supplementary material_P2Y13 Meta Analysis.docx]

International Journal of Molecular Sciences

Characterization of Potency of the P2Y13 Receptor Agonists: A Meta-Analysis

Chrisanne Dsouza ^1,2^ and Svetlana V Komarova ^1,2,3,^*

^1^ Department of Experimental Surgery, McGill University, Montreal, QC H3G 1A4, Canada;
chrisanne.dsouza@mail.mcgill.ca

^2^ Shriners Hospital for Children, Montreal QC H4A 0A9, Canada

^3^ Faculty of Dentistry, McGill University, Montreal, QC H3A 1G1, Canada

* Correspondence: [svetlana.komarova@mcgill.ca](mailto:svetlana.komarova@mcgill.ca)

**Supplemental data**

**S1**. Search strategy for the P2Y13 meta-analysis

| **Search strategy:** | |
| --- | --- |
| Database | PubMed |
| Search date | initial: July 03, 2019; updated: September 14, 2020 |
| Search strategy | (“P2RY13” OR ”GPCR1” OR ”GPR86” OR ”GPR94” OR ”P2Y13” OR ”SP174” OR ”purinergic receptor P2Y13” OR “53829” OR “74191”) AND (“ATP” OR “adenosine triphosphate” OR “ADP” OR “adenosine diphosphate” OR “UTP” OR “uridine triphosphate” OR “UDP” OR “uridine diphosphate” OR “[33P]2MeSADP” OR “2MeSADP” OR “2MeSATP” OR “ADPβS” OR “ATPγS” OR “cangrelor” OR “Ap4A” OR “MRS2603” OR “MRS2211” OR “reactive blue-2” OR “suramin” OR “2MeSAMP” OR “PPADS”) |
| Eligibility criteria | P2Y13 response to P2Y13-specific ligands |
| Initial hits | 153 |
| Eligible | 50 |
| Potential datasets | 18 |

**S2**. Quality assessment questionnaire for the articles included in the meta-analysis. 14 points indicates a high-quality article

| **Quality checklist (maximum 14 points):** | |
| --- | --- |
| 1 | Whether the study focused specifically the P2Y13 (receptor overexpression or specific agonist) (1) or other general agonists were added which can generate a response through other P2 receptors (0) |
| 2 | Whether the study reported the following: (1 point each, maximum of 8): dose dependence curves, agonist/antagonist addition, reporting concentration of ligand, expression of receptor, type of assay, outcome, sample size, origin of cells (in vivo, in vitro, etc.) |
| 3 | Whether the study reported control data (1) or normalized to control (1) |
| 4 | Whether data was presented in a graph (1) or tabular form (0) |
| 5 | When only EC_50_ was reported (0), or a dose dependency curve was provided (1) |
| 6 | Whether the paper reported what was the measure of central tendency (e.g. mean, median) or spread (e.g. SD or SE) (1) or whether it was unclear (0) |
| 7 | Contains detailed information on whether cells were exposed to particular treatments (1) (CP/CPK, other antagonists etc.) or if some aspects were unclear (e.g. duration, concentration, application) (0) |

**S3**. Study level effect sizes (EC_50_/IC_50_) of the reported outcomes and subgroups in P2Y13-mediated events, and steepness (S) in the largest dataset of signaling outcomes (Ca^2+^/IP_3_ release)

*Please refer to the Excel document “Table S3- EC_50_ and steepness”*
